# Antioxidant and anti-inflammatory activities of lycopene against 5-fluorouracil-induced cytotoxicity in Caco2 cells

**DOI:** 10.1016/j.jsps.2022.09.011

**Published:** 2022-09-20

**Authors:** Norah M. Alhoshani, Mohammed Al-Zharani, Bader Almutairi, Nada H. Aljarba, Norah S. AL-Johani, Nora Alkeraishan, Abdullah A. AlKahtane, Saud Alarifi, Daoud Ali, Saad Alkahtani

**Affiliations:** a Department of Zoology, College of Science, King Saud University, P. O. Box 2455, Riyadh 11451, Saudi Arabia; b Imam Mohammad Ibn Saud Islamic University (IMSIU), College of Science, Biology Department, Riyadh 11623, Saudi Arabia; c Department of Biology, College of Science, Princess Nourah bint Abdulrahman University, P. O. Box 84428, Riyadh 11671, Saudi Arabia

**Keywords:** 5-fluorouracil, Lycopene, Cytotoxicity, Oxidative stress, Inflammation, 5FU, 5-fluorouracil, CC, colorectal cancer, L, lycopene, LDH, Lactate dehydrogenase assay, SOD, Superoxide dismutase, GSH, Glutathione, IL, Interleukin, iROS, intracellular reactive oxygen species

## Abstract

5-fluorouracil (5FU) is widely used to treat colorectal cancer (CC) and its main mechanisms of anticancer action are through generation of ROS which often result in inflammation. Here, we test the effect of Lycopene against 5FU in Caco2 cell line. Caco2 cells were exposed to 3 µg/ml of 5FU alone or with 60, 90, 120 µg/ml of lycopene. This was followed by assessment of cytotoxicity, oxidative stress, and gene expression of inflammatory genes. Our findings showed that Lycopene and 5FU co-exposure induced dose-dependent cytotoxic effect without compromising the membrane integrity based on the LDH assay. Lycopene also significantly enhanced 5FU-induced SOD activity and GSH level compared to control for all mixture concentrations (*p* < 0.01). Lycopene alone and combination with 5FU-induced expression of *IL-1β, TNF-α*, and *IL-6*. Furthermore, *IFN-γ* expression was significantly enhanced by only mixture of lycopene (90 µg/ml) and 5FU (*p* < 0.05). In conclusion, Lycopene supplementation with 5FU therapy resulted in improvement in antioxidant parameters such as catalase and GSH levels giving the cell capacity to cope with 5FU-mediated oxidative stress. Lycopene also enhanced IFN-γ expression in the presence of 5FU, which may activate antitumor effects further enhancing the cancer killing effect of 5FU.

## 1 Introduction

One of the most common forms of cancer that significantly impacts global burden of cancer deaths is colorectal cancer (CC). Being the third on global cause of mortality, prognosis for colorectal cancer still remains unpredictable for more than 50 % of patients affected with the cancer (Ferlay et al., 2015, Sung et al., 2021). Primarily, availability of chemotherapeutic agents and advancement in treatment modalities have significantly improved patients overall survival for CC especially for those in early disease stages. However, patients with advanced stage disease with metastasis have considerably poor prognosis for overall survival because conventional therapies are unsuitable for treating metastatic tumor cells (Simon, 2016).

One major problem with chemotherapy in cancer treatment is the associated toxicity despite the significant increase in the patients’ overall survival. Oxidative stress because of reactive oxygen species (ROS) is a major factor responsible for CC pathogenesis (Basak et al., 2020). Generated ROS includes hydroxyl radicals (HO^•^), superoxides (O^2•−^) and hydrogen peroxide (H_2_O_2_). These ROS mediate genetic alterations due to DNA oxidation resulting in DNA damage that may be critical to propagation and progression of cancers like CC (Bazhin et al., 2016). ROS can oxidize DNA bases causing lesions that create either a single or double strand breaks. When such modifications occur in genes of important proteins such as the tumour suppressor, p53 protein, this is the pathogenesis of cancer development.

5FU is a conventional drug that is widely investigated and it is the first line treatment of choice for patients with CC (Peng et al., 2020). One of the mechanisms of anticancer action of 5FU is the generation of ROS such as HO^•^ and O^2•−^ which attach cancer cells in several different ways. Furthermore, these surge in intracellular ROS generation by 5FU is the main fator responsible for the major side effects associated with 5FU therapy such as cardiotoxicity, hepatotoxicity and nephrotoxicity (Refaie et al., 2021, Elghareeb et al., 2021). This is especially because this mechanism of action is not target specific as seen in immunotherapy, thus affecting nearby normal healthy cells. Several studies have investigated the use of antioxidants, specifically dietary antioxidants, to suppress the side effects of 5FU (Rtibi et al., 2021). One such useful natural antioxidant that has been widely investigated to suppress chemotherapy induced side effects of drugs such as cisplatin is lycopene (Kulhan et al., 2019). Lycopene is a carotenoid compound that is abundantly present in tomatoes, guava and watermelon (Moroni et al., 2021). There is well-established report within the literature documenting the antioxidant activity of lycopene in ameliorating chemotherapy induced toxicity. Moroni et al. (2021) showed that lycopene suppressed skin toxicity induced by panitumumab in patients with metastatic CC. Furthermore, lycopene was found to significantly suppress inflammatory responses in CC cells by inhibiting pro-inflammatory cytokines expression like cyclooxygenase-2 (COX-2), interleukin 1β (IL-1β), IL-6 and tumor necrosis-α (TNF-α), (Cha et al., 2017). Here, we investigated the role of lycopene in mediating antioxidant and anti-inflammatory effects against 5FU mediated oxidative stress and inflammatory responses.

## 2 Methodology

### 2.1 Cytotoxicity assay

The cytotoxic effect of both drug 5FU and Lycopene on colorectal cancer cell line was evaluated in Caco2 using MTT assay. Cells were grown and then exposed to different concentration of 5FU and Lycopene for 48 hrs. The IC50 value were determined for each drug and was 6.1 µg/ml for 5FU and 183.7 µg/ml for lycopene (Alhoshani et al., 2022).

### 2.2 Lactate dehydrogenase assay (LDH)

LDH assay (Sigma Aldrich, St Louis, USA) was employed to assess cell viability. Cells were exposed to 3 µg/ml of 5-FU; 60, 90, 120 µg/ml of Lycopene, and mix of the two compounds (3 µg/ml of 5-FU plus 60 µg/ml of lycopene), (3 µg/ml of 5-FU mixed with 90 µg/ml of lycopene), (3 µg/ml of 5-FU plus 120 µg/ml of lycopene). A 100 µl of culture medium without cells were collected and kept on ice after 24 h exposure. Mixture of 50 µl of sample culture medium and 50 µl of master mix (2 µl LDH substrate and 48 µl assay buffer) added per well. In new 96 well plate and incubated as above with shaking for 15 mins following absorbance measurement at 450 nm.

### 2.3 Superoxide dismutase (SOD)

Cells were seeded at density 1 × 10^6^/ml, with 24 h incubation. Afterwards, the cells were then treated as follows: 3 µg/ml of 5FU; 60, 90, 120 µg/ml of Lycopene, and mix of two compounds as (3 µg/ml of 5-FU plus 60 µg/ml of lycopene), (3 µg/ml of 5-FU mixed with 90 µg/ml of lycopene), (3 µg/ml of 5-FU plus 120 µg/ml of lycopene), then incubated again for 24 h at 37 °C. After, the cells were collected and washed with 1x PBS then turn to lysate by using the cold buffer with sonicating for 5 min with 1 ml of (210 mM mannitol, 1 mM EGTA, 20 mM HEPES and 70 mM sucrose pH 7.2). Cells were centrifuged for 5 mins at 1500 rpm and 4 °C to collect the supernatant. The SOD assay (Cayman SOD kit No.706002) was performed by mixing 200 µl diluted radical detector with 10 µl sample per well of 96-well plate. Reaction initiation was done by 20 µl diluted Xanthine Oxidase in each well, and followed by 30 mins incubation at room temperature with shaking. Absorbance scan was done at 440–460 nm range. SOD activity was evaluated by:

SOD = [(sample LR − Y − (intercept/slope)) × 0.23 ml/ 0.01 ml] × sample dilution.

### 2.4 Glutathione (GSH)

1 × 10^6^/ml of cell was seeded with incubation for 24 h at 37 °C. Afterwards, cells were exposed the above then incubated again for 24 h at 37 °C. The cells were detached by using scrapper and washed in 1X PBS then sonicated for 5 mins with 1 ml of PBS. After sonication, the cells were then centrifuged 1500 rpm for 5 mins at 4 °C. Collected supernatant was transferred into new 1.5 ml tube. The GSH assay (Cayman GSH kit No.703002) was using manufacturer’s instructions. A 50 µl of sample and 150 µl of cocktail 11.25 ml (2-(*N*-morpholino) ethanesulfonic acid MES Buffer, were mixed and 0.45 ml reconstituted to a cofactor, 2.1 ml reconstituted enzyme, 2.3 ml H_2_O, and 0.45 ml of 5,5′-dithio-bis-2-nitrobenzoic acid. The plate was incubated for 25 mins in the dark, and absorbance measured at 410 nm.

### 2.5 Catalase activity

A 1 × 10^6^ cells /ml was exposed to the drugs as above then incubated again for 24 h at 37 °C. After incubation, the cells were rinsed with 1X PBS then sonicated in catalase assay buffer for 5 min. The cells were then centrifuged at 4 °C, 10 mins at 10000 rpm, and supernatant was collected. Catalase assay (Bio vision, Catalog No. K773-100) was performed by adding 2 µl of sample to assay buffer up to 78 µl in each well of 96 well plate and incubated for 5 min at 25 °C. Afterwards, 50 µl developer mix (46 µl catalase assay buffer, 2 µl OxiRed ^TM^ Probe, 2 µl HRP lyophilized solution) was added per well and incubated at 25 °C for 10 mins and absorbance measurement done at 570 nm.

### 2.6 Intracellular ROS generation

ROS generation was analyzed by using ROS assay Sigma- Aldrich (St Louis, MO, USA). Cells were grown on 96 well black microplate as well as 6 well plate at 5 × 10^5^ cells/ml in 10 % cultured medium incubated for 24 h at 37 °C. Afterwards, cells were treated as previously and incubated for 24 h. The media was aspirated, then 100 µl of new media was mixed with 5X concentration of deep red solution to per well of the 96 well plate while a 1 ml of same solution was added per well of 6 well plate. After addition, cells were incubated for an hour at 37 °C. Absorbance was then read at 485–528 nm range. For imaging, the media was aspirated from the 6 well plate, cells were washed several times with 1X PBS and imaged under fluorescence microscope (DMLB, Leica, Germany).

### 2.7 Gene expression

Analyses of *TNF-α, IL-1α, IL-1β, IL-6, IL-27, IL-33, INF-γ*, *Cox-1*, and *Cox-2* mRNA levels were done by RT-PCR (PE Applied Biosystems, Foster City, California). Briefly, Caco2cells were treated as previously and incubated as above. Total RNA was extracted with TRIzol RNA Reagent (Cat.No.79306) then converted into cDNA by utilizing cDNA kit, (Thermofisher Cat. No. 4368814. USA). A 1000 ng of purified RNA sample was used to prepare the complementary single strand of cDNA. The total volume of one cDNA reaction was 20 µl containing10 µl of master cDNA mix + 10 µl of purified RNA). After CDNA synthesis, 2.5 µl of cDNA was mixed with 17.5 µl of Go Taq qPCR Master Mix (SYBR® green). The master mix of each sample were prepared as follows; 10 µl of Go Taq Green Master, 0.8 µl of forward β- actin, 0.8 µl of reverse β-actin, and 5.9 µl of Nuclease- free water. The fold change level determination for target gene including the exposure and non-exposure cells was calculated using the ΔΔCT method as follows: Normal expression ratio = 2e^−ΔΔCT^ [where ΔCT = Ct (target) − Ct (β-actin) and ΔΔCt = ΔCt (treated sample) − ΔCt (Untreated sample)].

## 3 Results

Based on determined IC50 for drugs, 3 µg/ml of 5FU and 60, 90, 120 µg/ml of lycopene were selected for further experiments.

### 3.1 Effect on cell membrane integrity

**Fig. 1:**
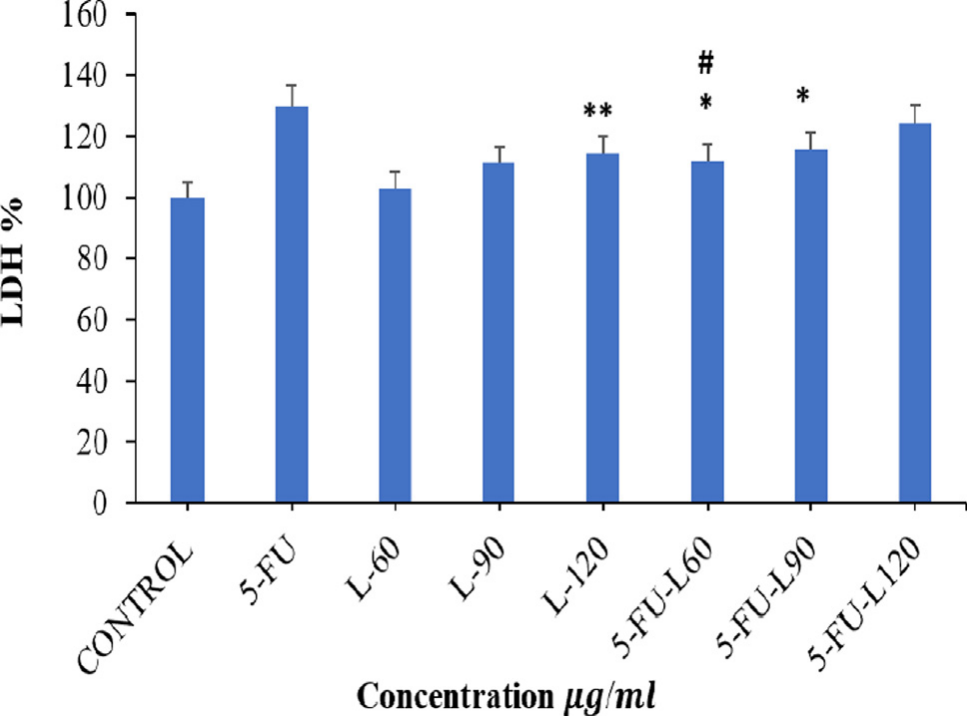
Effect of Lycopene, 5FU, different mixtures of both on cell viability of Caco2 cancer cell line by using LDH assay. Data represents mean ± SD where * p < 0.05 and ** p < 0.01 shows significance compared to control and # p < 0.05 indicates significant effect of mixed drugs compared to 5FU.

LDH is an oxidoreductase enzyme catalyzing conversion of pyruvate into lactate. In pathological condition such as cancer or damaged tissue, cell release LDH into bloodstream due to damage to the plasma membrane, implying toxic effect of cell damaging compounds. Caco2 cells were exposed to 3 µg/ml of 5FU; 60, 90, 120 µg/ml of Lycopene, mix of 3 µg/ml of 5FU with either 60, 90, or 120 µg/ml of Lycopene for 24 h. Assessment of the toxic effect of these compounds as evaluated by the LDH assay indicated that 120 µg/ml of Lycopene as well as mixture 3 µg/ml of 5FU and either 60 or 90 µg/ml of Lycopene mixed induced significant toxicity on the cell line compared to control. However, 60 µg/ml of Lycopene seem to suppress 5FU induced toxicity (*p* < 0.05) (Fig. 1).

### 3.2 Generation of ROS

**Fig. 2:**
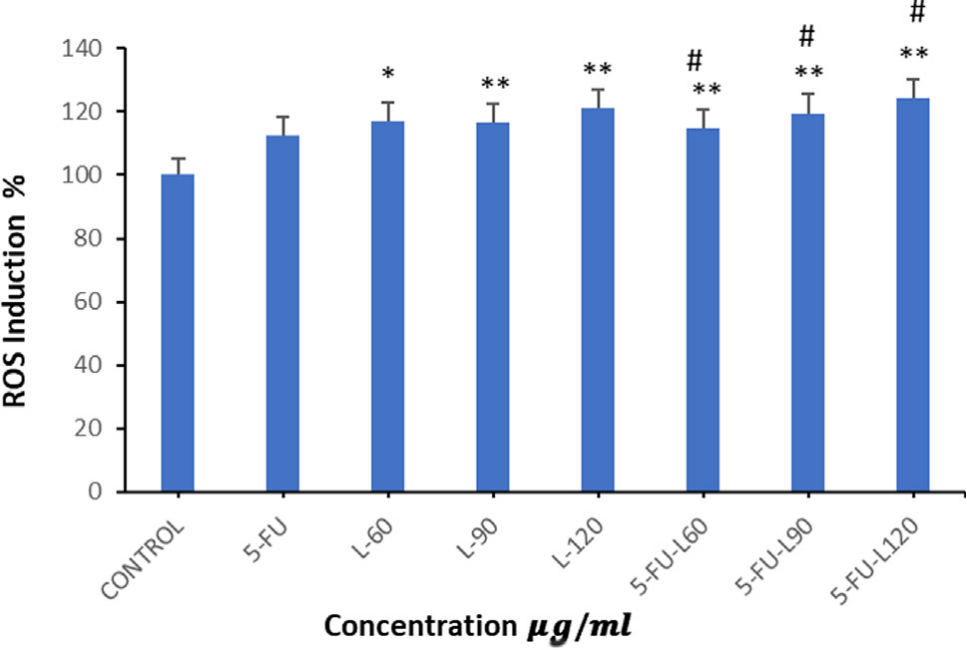

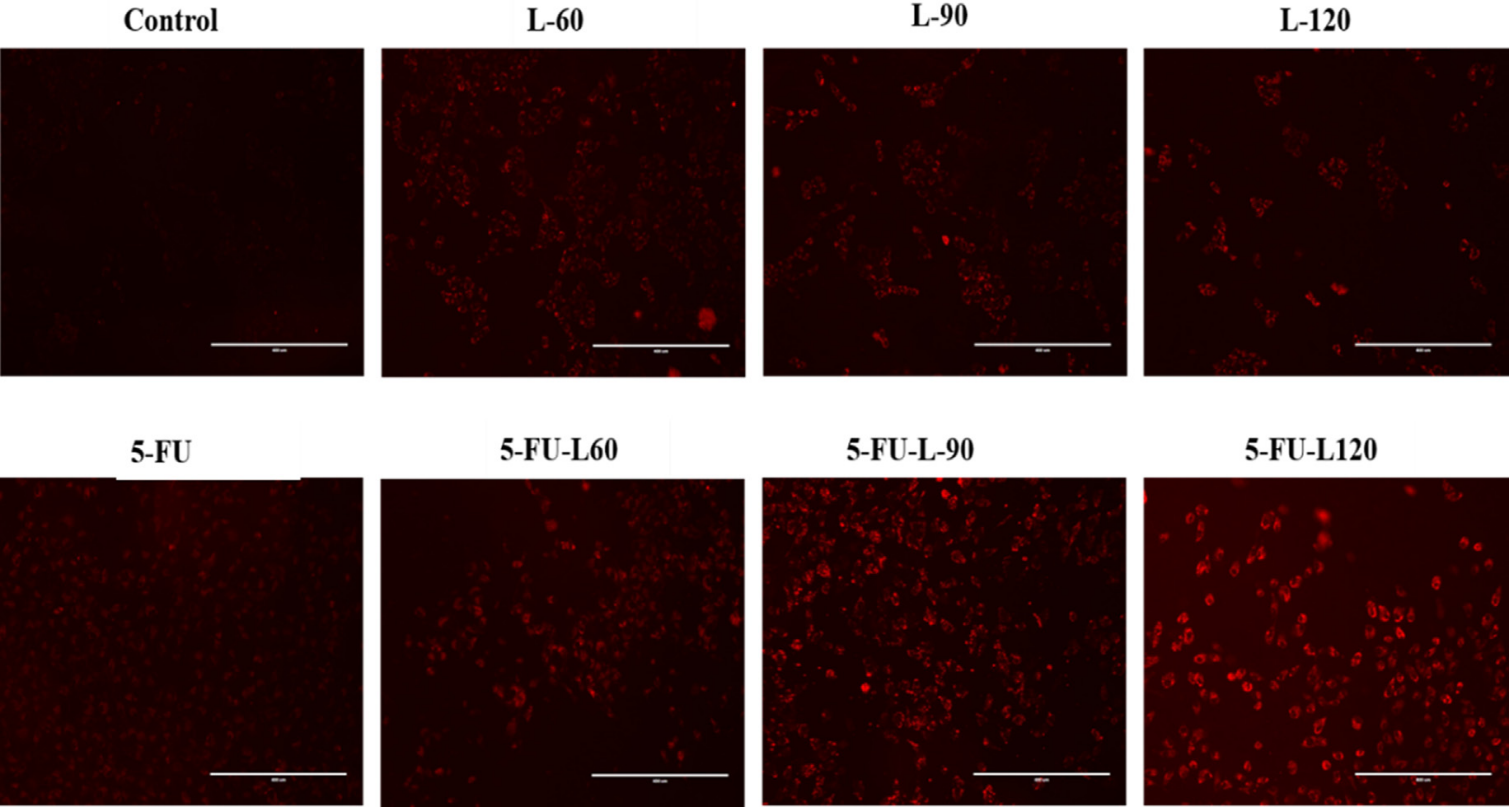
**A**. Increased of ROS level 24 h exposure to Lycopene, 5FU, different mixtures of both in Caco2 cancer cell line. Data represents mean ± SD where * p < 0.05 and ** p < 0.01 shows significance compared to control and # p < 0.05 indicates significant effect of mixed drugs compared to 5FU. **B**. ROS generation after 24 h exposure to Lycopene, 5FU, different mixtures of both in Caco2 cancer cell line, (1. control), (2. 5FU), (3. L60), (5. L90), (7.L120), (4. 5FU-L60), (6. 5FU-L90), (8. 5FU-L120).

Caco2 cell line were exposed to varying concentrations of 5FU, lycopene and mixture of both as outlined earlier to evaluate the impact of these exposure on generation of intracellular ROS. Our results indicated the cells treatment with 60 µg/ml lycopene significantly increased ROS generation (* *p* < 0.05), as well as the L-90, L-120, 5FU-L-60, 5FU-L90, and 5FU-L120. Furthermore, L60 and L120 seemed to enhance 5FU-induced ROS generation (Fig. 2A & B).

### 3.3 Activity of oxidative stress markers

**Fig. 3:**
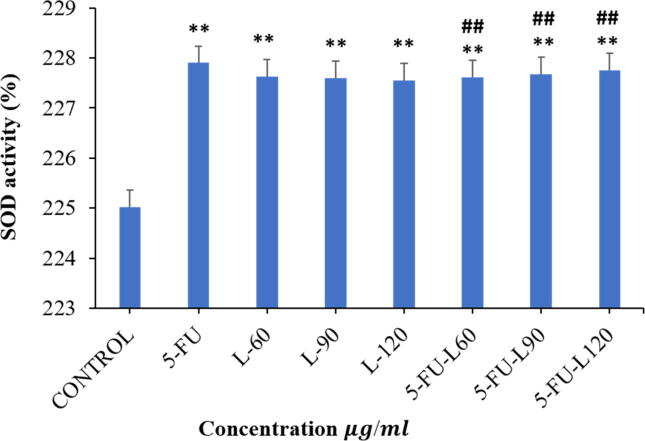
Effect of Lycopene, 5FU, mix of them in increasing the activity of SOD protein in Caco2 cancer cell line post-24 h exposure to drugs. Data represents mean ± SD where * p < 0.05 and ** p < 0.01 shows significance compared to control and # p < 0.05 and ## p < 0.01 indicates significant effect of mixed drugs compared to 5FU.

Superoxide dismutase (SOD) is a metalloenzyme facilitates the disassociation of superoxide anion into oxygen molecule and hydrogen peroxide. This disassociation process is one of the various cellular anti-oxidant defense systems protecting the cell from oxidative damages (Cecerska-Heryć et al., 2021). Exposure of Caco2 cell line to different concentrations of Lycopene and/or 5FU to understand influence of these compound on dissipation of oxidants showed that there was a significant increase SOD activity for all treatments compared to control (*p* < 0.01) while 5FU-L60, 5FU-L90, and 5FU-L120 increased 5FU mediated suppression of 5FU-induced SOD activity (*p* < 0.01) (Fig. 3).

**Fig. 4:**
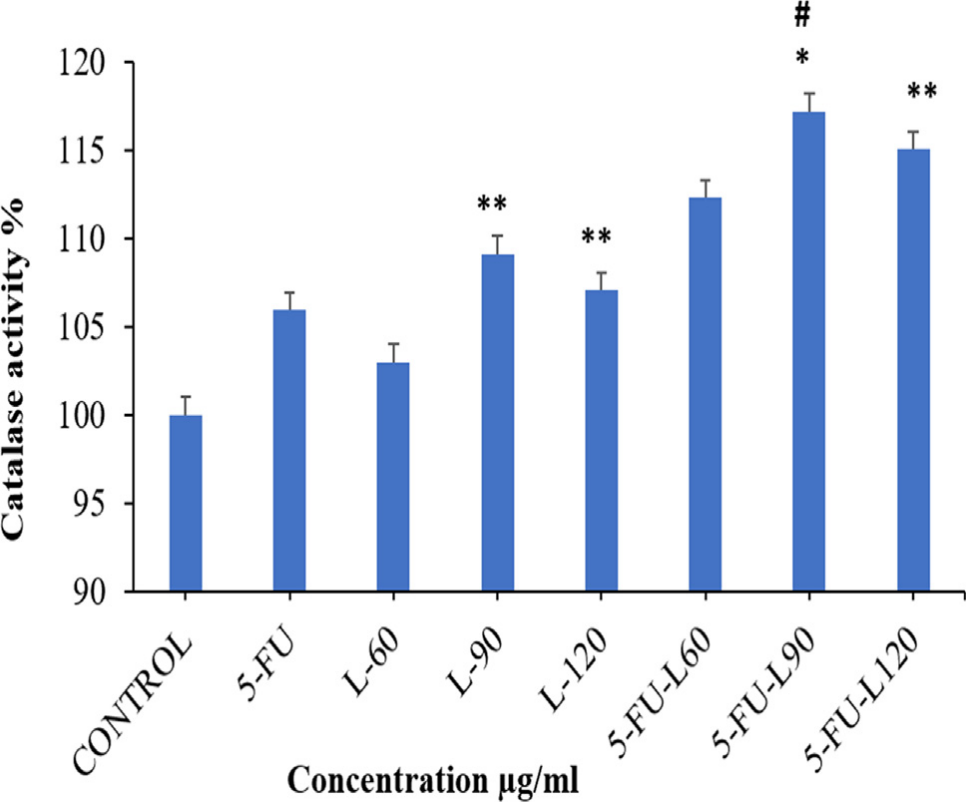
Increasing the catalase activity after 24 h exposure with Lycopene, 5FU, mix of them in Caco2 cell line. Data represents mean ± SD where * *p* < 0.05 and ** *p* < 0.01 shows significance compared to control and # *p* < 0.05 indicates significant effect of mixed drugs compared to 5FU.

Catalase is an intracellular enzyme that enhance the disassociation of H_2_O_2_ into H_2_O and O_2_ in order to suppress associated oxidative stress (Tehrani and Moosavi-Movahedi, 2018). Caco2 cell line that were exposed to L90 and L120 with or without 5FU were found to significantly increase catalase activity (*p* < 0.01, *p* < 0.05) (Fig. 4). Furthermore, L90 also significantly increased catalase activity compared to 5FU alone (*p* < 0.05).

**Fig. 5:**
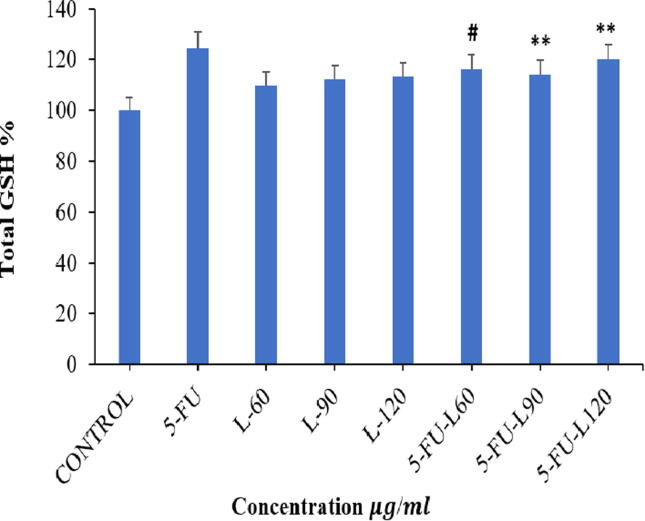
Assessment of GSH levels 24 h post-exposure of Caco2 cell line to Lycopene, 5FU, mixtures of both. Data represents mean ± SD where * p < 0.05 and ** p < 0.01 shows significance compared to control and # p < 0.05 indicates significant effect of mixed drugs compared to 5FU.

Reduced glutathione (GSH) is a tripeptide that functions in scavenging of many reactive species either within or outside of the cell (Schmidt & Dringen., 2012). Because of the ROS levels, SOD and catalase activities, the level of GSH in Caco2 cells was evaluated after exposure to the drugs. The results show a significant increase in cells exposed to 5FU-L60 when compared with 5FU (*p* < 0.05). Similarly, 5FU-L90 and 5FU-L120 also induced significant increase in GSH levels in exposed cells compared to control untreated cells (*p* < 0.01) (Fig. 5).

### 3.4 Expression of inflammatory markers

**Fig. 6:**
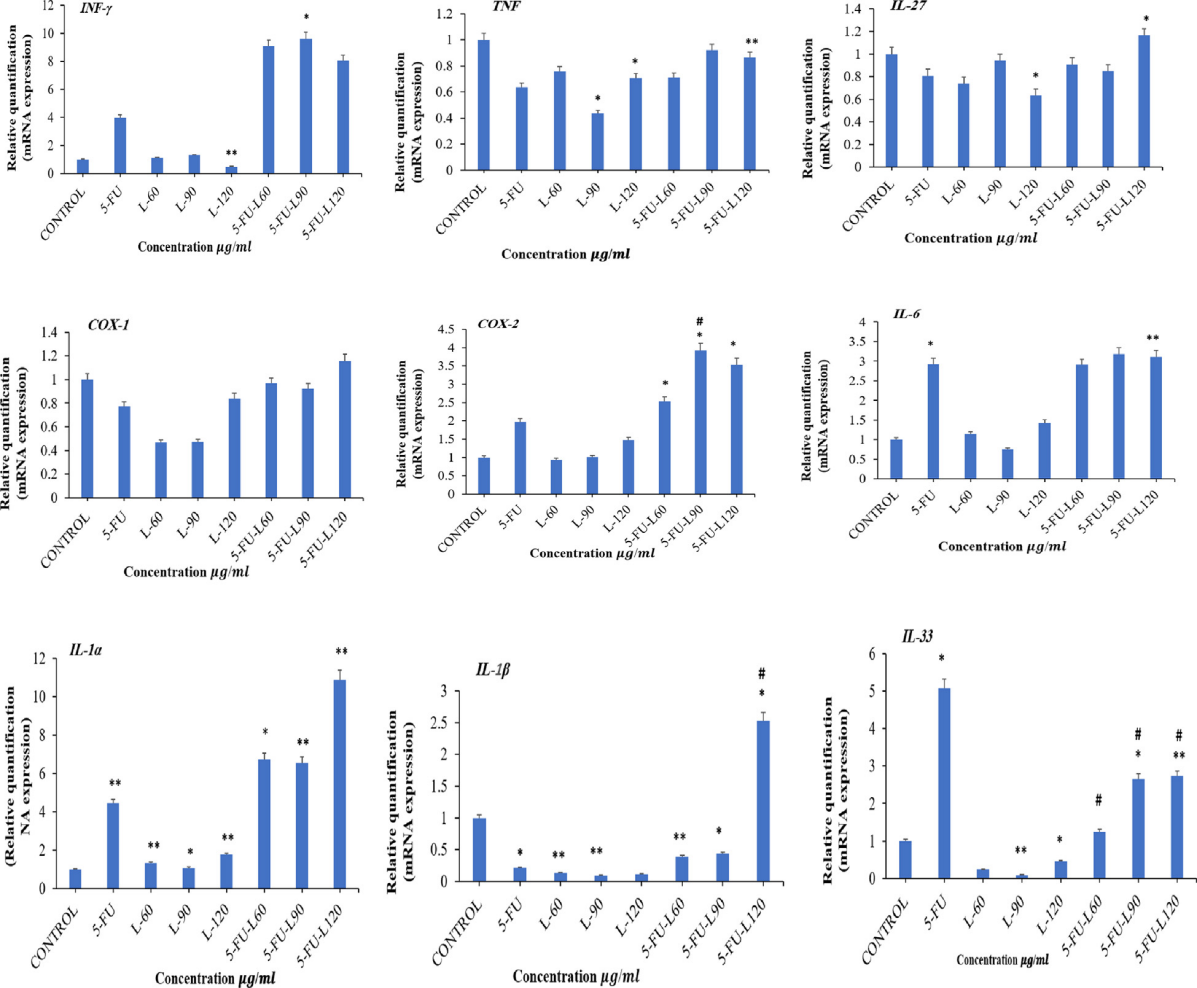
Effect of Lycopene, 5FU, mixtures of both on INF-γ, TNF-α, IL-27, Cox-1, Cox-2, IL-6, IL-1α, IL-1β, and IL-33 mRNA expression in Caco2 cancer cell line. Data represents mean ± SD where * p < 0.05 and ** p < 0.01 shows significance compared to control and # p < 0.05 indicates significant effect of mixed drugs compared to 5FU.

Because oxidative stress is tightly associated with inflammatory responses, we evaluated the gene expression of different pro-inflammatory cytokines (Fig. 6). Analysis of *INF-γ* expression showed that 5FU-L90 significantly increased the gene expression (*p* < 0.05) whereas a treatment with L120 significantly reduced the expression of INF-γ gene (*p* < 0.01). *TNF-α* expression was found to significantly decrease after 24 h exposure to L-90, L-120 and 5FU-L120 (*p* < 0.05, and *p* < 0.01). Furthermore, all other exposures resulted in a considerable but non-significant decrease in the gene expression of TNF-α. Assessment of *IL-27* expression in the Caco2 cell line showed that L-120 exposure resulted in significant decrease in *IL-27* (*p* < 0.05). Contrastingly, co-exposure of the cells to 5FU and L120 caused a significant increase of *IL-27* expression (*p* < 0.05). Cox-1 and Cox-2 are involved in prostaglandins syntheses from arachidonic acid and their expression is known to be linked to oxidative stress. Exposure of Caco2 cells to all single exposures of the compounds or combinations did not significantly influence *Cox-1* expression. However, considerable reduction in *Cox-1* expression was found upon exposure to 5FU, L-60 and L90 while combination of 5FU and L120 slightly increased *Cox-1*. As for *Cox-2*, 5FU-L60, 5FU-L 90, and 5FU-L120 significantly increased *Cox-2* expression compared to control (*p* < 0.05). Furthermore, the exposure with 5FU-L-90 has also a significant increase in *Cox-2* expression (*p* < 0.05) compared to 5FU. Other treatment 5FU and Lycopene has not any significant effect in *Cox-2* expression. Assessment of *IL-6* expression indicated that 5FU significantly increased expression of the gene (*p* < 0.05) and similarly for cells exposed to 5FU-L-120 (*p* < 0.01) compared to control. All single exposures to Lycopene were found to not influence *IL-6* expression even at the higher dose of L120. In a similar trend for cells exposed to 5FU, there was significant increase in *IL-1α* expression while exposure to all single doses of Lycopene resulted in significant reduction in *IL-1α* expression compared to control group (*p* < 0.01, *p* < 0.05). However, it was found that combination of 5FU and all doses of Lycopene causes an increase in the gene expression when compared to control. We also found that L120 significantly increased 5FU-mediated expression of *IL-1α* (*p* < 0.05).

All exposures resulted in significant reduction in *IL-1β* expression compared to control (*p* < 0.05) except for combinational exposure to 5FU-L120 which significantly increased *IL-1β* expression (*p* < 0.05) compared with control as well as compared with 5FU (*p* < 0.05). Expression of *IL-33* in Caco2 cells showed that 5FU significantly increased *IL-33* expression while L90 and L120 exposures resulted in reduction in the gene expression. When compared with the control untreated cells, L90 and L120 significantly reduced *IL-33* expression while L60 did not influence expression of the gene when compared with control untreated cells. All the combination exposures caused a significant increase in *IL-33* expression except for 5FU and L60 when compared to control cells (*p* < 0.05, *p* < 0.01). Interestingly, all doses of Lycopene significantly reduced 5FU-mediated expression of *IL-33* (*p* < 0.05).

## 4 Discussion

In this study, we investigated co-exposure of lycopene with the conventional anticancer drug, 5FU to understand its influence as an antioxidant and anti-inflammatory on 5FU mediated responses on Caco2 cells. 5FU-mediated ROS generation is reported to be associated with its cytotoxic effects on both cancer and normal cells, which is one of the setbacks to the application of 5FU as anticancer drug (Blondy et al., 2020). The body’s anti-oxidant defense system is comprised of different factors such as enzymes like catalase and SOD or organic compounds like GSH. The balance between these factors and ROS or reactive nitrogen species within the cells dictates the development of oxidative stress or redox balance. Furthermore, the activities of these factors are dependent upon the rate of ROS or RNS generation within the cells.

Oxidative stress occurs because of the instability of generated ROS, which attacks intracellular molecules inside tumor microenvironment. There is high generation of ROS such as hydroxyl radical, hydrogen peroxide and superoxide which are common in CC and other cancers (Cheung and Vousden, 2022). One of 5FU mechanisms in CC, is the activation of apoptotic signals that is transmitted into the nucleolus (Pagliara et al., 2016). The reports that chemotherapy with 5FU result induction of ROS during cancer treatment, is supported by our finding in this study. This increased ROS generation activates the anti-oxidant defense increasing the activities of SOD and catalase, which catalyzes superoxide to hydrogen peroxides; and hydrogen peroxide to oxygen and water respectively (Das and Roychoudhury, 2014). However, chemotherapeutics like 5FU cause redox imbalance by suppressing the activities of catalase and SOD as well as reduce GSH levels during high ROS generation (Kocer and Naziroglu, 2013). This is similar to the finding in this study where 5FU resulted in no increase in GSH level and catalase activity, creating a scenario where there are low activities of antioxidant that can handle the increase in ROS generation. This scenario is characteristic of the redox imbalance required for its cell growth inhibitory action.

Lycopene is a known antioxidant which we employed here to enhance the cell killing effect of 5-FU with reduced inflammation and oxidative stress. Previous investigations have demonstrated the ability of Lycopene to inhibit toxicity of different anticancer drugs (Karimi et al., 2005, Kulhan et al., 2019, Moroni et al., 2021). While we found Lycopene to induce ROS generation in this cell line, combinational treatment of Lycopene and 5FU was also found to increase SOD and catalase activities in addition to an increase in GSH level which will help the cell maintain redox balance. Also in support of our finding, some studies have shown that Lycopene suppresses toxic effect of chemotherapeutics due to the antioxidant effects. Atessahin et al., 2006, Elsayed et al., 2021 showed that Lycopene respectively reduced testicular toxicity of induced by Adriamycin and hepatotoxicity induced by cisplatin through restoration of the depleted GSH level and reduction of malonaldehyde levels in rat models. However, Lycopene antioxidant activities appears to be a double-edged sword as a previous study have shown that the oxidant or antioxidant activities of Lycopene in Caco2 cell line is dependent on the concentration used (Makon-Sébastien et al., 2014). This may explain why we observed an increase in ROS generation in this study compared to some other stated earlier.

Oxidative stress and inflammation are linked. Inflammation is one of the body’s defense systems during pathogenic infection. Increased ROS generation during these disease states result in oxidation of biomolecules like proteins and lipids causing activation or expression of inflammatory signals like *COX1/2, IL-1β, IL-6* and *TNF-α* (Liu et al., 2017, Harijith et al., 2014). There is imbalance in immune response during low GSH levels and high ROS which aggravates inflammation that may drive cancer progression (Wu et al., 2014). One of the mechanisms used by cancer cells is to drive inflammation that result in chemo-resistance and this is a common phenomenon in 5FU (Wang et al., 2016). There are studies that have demonstrated that 5FU alters pro-inflammatory cytokines expression like *TNF-α, IL-1β, and IL-6* (Logan et al., 2008, Raghu Nadhanan et al., 2012, Reers et al., 2013). IL-6 in particular may drive cancer progression by activating the JAK/STAT pathway resulting in unending loop of IL-6 mediated inflammation (Yusuf and Casey, 2019, Hu et al., 2021). While we found that 5FU induced increased expression of *TNF-α, IL-1β, IL-1α* and *IL-6* which may drive tumorigenesis, all doses of Lycopene that we tested did not suppress the expression of these inflammatory cytokines. This may have been due to the concentration of Lycopene that was trialed in this study as we have highlighted earlier. As such adjusting these concentrations may help harness and improve Lycopene anti-inflammatory effect in the presence of 5FU. Interestingly, expression of *INF-γ* was enhanced by Lycopene in cells co-exposed to 5FU. IFN-γ is a cytokine known to have antitumor activity by activating cellular immunity, subsequently stimulating antitumor immune response (Jorgovanovic et al., 2020). Based on its anti-proliferative, pro-apoptotic, and cytostatic roles, IFN-γ has been highlighted to be potentially useful as an adjuvant immunotherapy in different cancer types.

## 5 Conclusion

We have shown here that Lycopene supplementation during 5FU therapy on Caco2 cell line resulted in improvement in antioxidant parameters such as catalase and GSH levels giving the cell capacity to cope with the oxidative stress mediated by 5-FU. We also showed that Lycopene enhanced *IFN-γ* expression in the presence of 5FU, which may activate antitumor effects further enhancing the cancer killing effect of 5FU. While Lycopene concentrations used in this study may have resulted in the increased inflammatory cytokines expression like *TNF-α, IL-1β,* and *IL-6*, adjustment of the concentration may further improve this anti-inflammatory role of Lycopene on 5FU-mediated inflammation.

## Availability of data and materials

The data generated or analyzed in this article are online publicly available without request.

## Authors' contributions

Norah Alhoshani and Nada Aljarba performed the cytotoxic assays. Mohammed Al-Zharani, and Bader Almutairi measured iROS. Saad Alkahtani, Norah S. AL-Johani and Daoud Ali performed genes expression. Saud Alarifi and Abdullah AlKahtane assessed catalase, GSH and SOD activities.

## Declaration of Competing Interest

The authors declare that they have no known competing financial interests or personal relationships that could have appeared to influence the work reported in this paper.
